# Infektion mit *Orientia tsutsugamushi* und nosokomiale Pneumonie

**DOI:** 10.1007/s00108-026-02185-3

**Published:** 2026-10-09

**Authors:** Ilka Grewe, Saskia Winzer, Silja Steinmann, Paul Francke, Aiman Gamal Abdelrahim, Annette Hennigs, Marylyn M. Addo, Michael Ramharter, Dominic Wichmann, Stefan Schmiedel

**Affiliations:** 1 I. Medizinische Klinik und Poliklinik, Universitätsklinikum Hamburg-Eppendorf, Hamburg, Deutschland; 2 Institut für Infektionsforschung und Impfstoffentwicklung (IIRVD), Zentrum für Innere Medizin, Universitätsklinikum Hamburg-Eppendorf, Hamburg, Deutschland; 3 Abteilung für klinische Immunologie von Infektionskrankheiten, Bernhard-Nocht-Institut für Tropenmedizin, Hamburg, Deutschland; 4 Standort Hamburg – Lübeck – Borstel – Riems, Deutsches Zentrum für Infektionsforschung, Hamburg, Deutschland; 5 Abteilung für Pneumologie, II. Medizinische Klinik und Poliklinik, Universitätsklinikum Hamburg-Eppendorf, Hamburg, Deutschland; 6 Abteilung für Klinische Forschung, Abteilung für Klinische Forschung, Hamburg, Deutschland; 7 Klinik für Intensivmedizin, Universitätsklinikum Hamburg-Eppendorf, Martinistr. 52, 20251 Hamburg, Deutschland

**Keywords:** Exanthem, Fieber unklarer Genese, Rickettsien-Infektionen, Scrub-Typhus, Tsutsugamushi-Fieber, Exanthema, Fever of unknown origin, Rickettsia infections, Scrub typhus, Tsutsugamushi-fever

## Abstract

**Hintergrund:**

Das Tsutsugamushi-Fieber ist eine durch *Orientia tsutsugamushi* verursachte, in Europa selten diagnostizierte Infektion, die in mehreren Ländern Asiens sowie in Chile endemisch ist. Das klinische Bild ist häufig unspezifisch und reicht von Fieber, Myalgien, Lymphadenopathie und einem makulopapulösen Exanthem bis hin zu schweren Verläufen mit Multiorganbeteiligung.

**Fallbericht:**

Ein 34-jähriger Mann stellte sich nach Rückkehr aus Thailand mit Fieber, Myalgien, Lymphadenopathie und einem makulopapulösen Exanthem vor. Trotz kalkulierter antiinfektiver Therapie kam es zu einer raschen klinischen Verschlechterung mit respiratorischer Insuffizienz, Vigilanzminderung und Hypotonie. Mit einer Polymerase-Kettenreaktions(PCR)-Analyse aus Vollblut konnte *Orientia tsutsugamushi *nachgewiesen werden. Unter Doxycyclin sowie Meropenem bei einer nosokomialen Pneumonie besserte sich der Zustand rasch.

**Schlussfolgerung:**

Dieser Fall unterstreicht, dass es wichtig ist, bei Reiserückkehrer:innen mit Fieber unklarer Genese an das Tsutsugamushi-Fieber zu denken. Eine zeitgerechte klinische oder molekulare Diagnostik sowie die rasche Einleitung einer wirksamen Therapie sind entscheidend, wenn schwere Verläufe mit Multiorganbeteiligung und sekundären Komplikationen verhindert werden sollen.

## Abstract (en)

**Background:**

Tsutsugamushi fever is an infection caused by *Orientia tsutsugamushi* that is rarely diagnosed in Europe but endemic in several Asian countries and in Chile. Its clinical presentation is often nonspecific, ranging from fever, myalgia, lymphadenopathy, and a maculopapular rash to severe disease with multiorgan involvement.

**Case report:**

A 34-year-old man presented with fever, myalgia, lymphadenopathy, and a maculopapular rash after returning from Thailand. Despite empiric antimicrobial therapy, his condition deteriorated rapidly, with respiratory failure, impaired consciousness, and hypotension. Polymerase chain reaction (PCR) testing of peripheral blood confirmed *O. tsutsugamushi*. Treatment with doxycycline, along with meropenem for hospital-acquired pneumonia, resulted in rapid clinical improvement.

**Conclusion:**

This case highlights the importance of considering tsutsugamushi fever in febrile travelers returning from endemic regions. Timely presumptive or PCR-based diagnosis and initiation of effective therapy are crucial to prevent severe disease with multiorgan involvement and secondary complications.

## Anamnese

Im August 2025 stellte sich ein 34-jähriger Mann mit Fieber bis 40,0 °C in einem Akutkrankenhaus in Oldenburg vor. Zehn Tage vor Aufnahme war er von einer zweiwöchigen Reise durch Thailand zurückgekehrt, die von Koh Samui über Koh Tao und Koh Phi Phi führte, bevor sie mit einem kurzen Aufenthalt in Chiang Mai endete. Während der Reise hielt sich der Patient sowohl in ländlichen als auch in urbanen Regionen auf, hatte außer dem Streicheln von streunenden Hunden keinen Kontakt zu Tieren, keine Verletzungen und keine Sexualkontakte. Drei Tage nach seiner Rückkehr nach Deutschland habe der Patient plötzlich ausgeprägte Abgeschlagenheit sowie generalisierte Myalgien und Fatigue entwickelt. Im Verlauf kam ein makulopapulöses Exanthem an Armen und Rücken hinzu, das von ihm initial als Exazerbation seiner bekannten atopischen Dermatitis interpretiert wurde. Abgesehen von der atopischen Dermatitis und einem Asthma bronchiale waren keine weiteren Vorerkrankungen bekannt.

## Untersuchung

Der Patient präsentierte sich in deutlich reduziertem Allgemeinzustand und schlankem Ernährungszustand. Der Patient war zu allen Qualitäten orientiert, ein Meningismus bestand nicht. Auskultatorisch waren keine Rasselgeräusche zu hören. Das Abdomen präsentierte sich leicht gespannt, jedoch ohne Zeichen eines Peritonismus. An den Armen und am Rücken präsentierte sich ein makulopapulöses Exanthem.

## Diagnostik

Laborchemisch zeigte sich initial eine geringe Erhöhung der Leukozytenzahl auf 10,6 Mrd./l sowie eine Erhöhung des C‑reaktiven Proteins (CRP) auf 124 mg/l und eine Erhöhung der Laktatdehydrogenase (LDH) auf 445 IU/l. In der Mikroskopie eines dicken Tropfens konnten wiederholt keine Plasmodien nachgewiesen werden. Ein Dengue-Antigentest und eine serologische Untersuchung auf Dengue-Fieber, ein Legionellen-Antigentest aus dem Urin, eine Polymerase-Kettenreaktions(PCR)-Analyse auf *Giardia lamblia* und eine PCR auf *Entamoeba histolytica* aus dem Stuhl, eine PCR auf Leptospiren aus dem Blut und ein Human-immunodeficiency-virus(HIV)-Antigen/Antikörper-Suchtest waren negativ. Blutkulturen blieben steril. In der Röntgenuntersuchung des Thorax waren keine Infiltrate zu sehen. Eine Sonographie des Abdomens zeigte eine geringe Splenomegalie, die später in einer Computertomographie des Thorax und Abdomens bestätigt wurde. Darüber hinaus zeigten sich in der Computertomographie multiple vergrößerte Lymphknoten paraaortal, mesenterial und axillär.

## Therapie und Verlauf

Der Patient wurde im Akutkrankenhaus in Oldenburg stationär aufgenommen. Es wurde eine antiinfektive Therapie mit Ampicillin/Sulbactam begonnen, die bei Progredienz des makulopapulösen Exanthems und Verdacht auf eine mögliche Arzneimittelassoziation wieder beendet wurde. Trotz anschließender Therapie mit Ceftriaxon (2000 mg intravenös 1‑mal/Tag) verschlechterte sich der klinische Zustand des Patienten kontinuierlich, einschließlich der Entwicklung einer Vigilanzminderung, einer progredienten respiratorischen Insuffizienz sowie eines zirkulatorischen Schocks, sodass eine Intubation mit anschließender invasiver Beatmung und eine Therapie mit Katecholaminen und Hydrokortison erforderlich wurden. Der Patient wurde daher auf die Intensivstation (ITS) verlegt und anschließend auf die ITS unseres Universitätsklinikums überführt, wo die kalkulierte antimikrobielle Therapie auf Meropenem (1000 mg intravenös 3‑mal/Tag), Vancomycin (500 mg intravenös 4‑mal/Tag) und Azithromycin (500 mg intravenös 1‑mal/Tag) umgestellt wurde. Eine erneute Computertomographie des Thorax zeigte Konsolidierungen beider Unterlappen mit einem positiven Bronchopneumogramm, passend zu pneumonischen Infiltraten. In bronchoskopisch gewonnenem Bronchialsekret wurden keine Erreger nachgewiesen.

Aufgrund der Vigilanzminderung erfolgte eine Liquorpunktion mit dem Befund von 38 Leukozyten/µl und einem Gesamteiweiß von 1962 mg/l. Die PCR-Diagnostik aus dem Liquor ergab einen negativen Befund für Herpes-simplex-Virus (HSV), Varizella-Zoster-Virus (VZV), *Haemophilus influenzae, Listeria monocytogenes, Neisseria meningitidis, Streptococcus agalactiae* und *Streptococcus pneumoniae*.

Zum Ausschluss einer malignen Genese sowie einer hämophagozytischen Lymphohistiozytose (HLH) erfolgte zudem eine Knochenmarkspunktion und die chirurgische Entnahme zweier vergrößerter axillärer Lymphknoten. Die histopathologische Untersuchung von Liquor, Knochenmark und der operativ exzidierten axillären Lymphknoten zeigte jeweils unspezifische reaktive Veränderungen ohne Hinweise auf Malignität oder HLH.

**Abb. 1:**
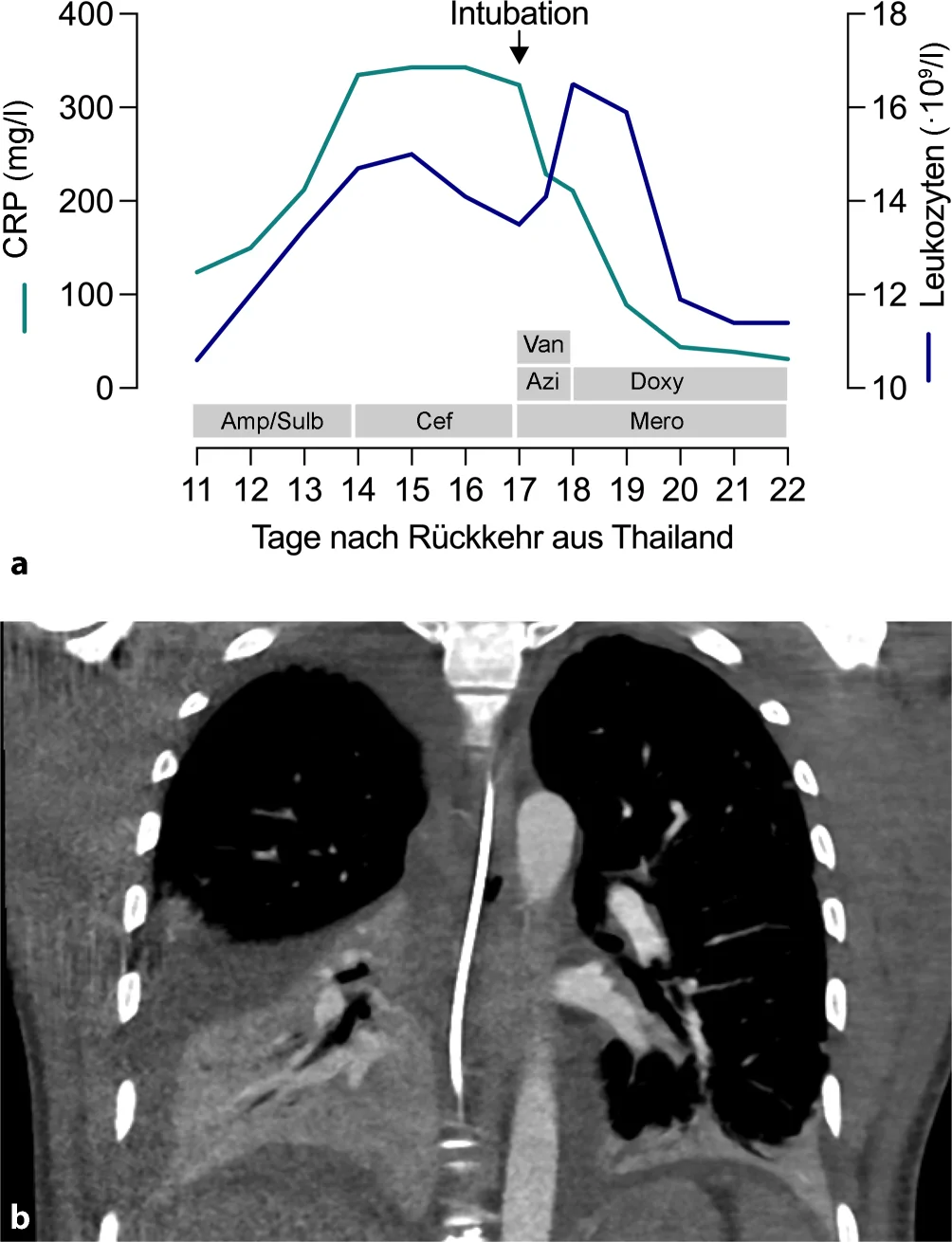
**a** Zeitverlauf von C‑reaktivem Protein (CRP) und Leukozytenzahl während der antiinfektiven Therapie mit Ampicillin/Sulbactam (Amp/Sulb), Ceftriaxon (Cef), Meropenem (Mero), Vancomycin (Van), Azithromycin (Azi) und schließlich Doxycyclin (Doxy) nach positiver Polymerase-Kettenreaktions-Analyse für *Orientia tsutsugamushi*. **b** Computertomographie mit Konsolidierungen in beiden Unterlappen

Schließlich konnte das Gen des bakteriellen 56 kDa-Antigens von *Orientia tsutsugamushi* mittels PCR im peripheren Blut nachgewiesen werden. Darüber hinaus konnte sowohl Immunglobulin M (IgM) als auch Immunglobulin G (IgG) gegen *Orientia tsutsugamushi* in einem indirekten Immunfluoreszenztest detektiert werden. Daraufhin wurde eine gezielte Therapie mit Doxycyclin (100 mg intravenös 1‑mal/Tag) eingeleitet und die zuvor begonnene Therapie mit Meropenem bei Verdacht auf eine zusätzliche nosokomiale Pneumonie fortgeführt. In der Folge normalisierten sich die Laborparameter (Abb. 1), der klinische Zustand besserte sich rasch und der Patient konnte drei Tage später extubiert und anschließend auf die Normalstation verlegt werden.

## Diskussion

*Orientia tsutsugamushi* zählt zur Familie der Rickettsiaceae und ist ein obligat intrazelluläres, gramnegatives Kurzstäbchen, das in Korea, China, Vietnam, Taiwan, Japan, Pakistan, Indien, Sri Lanka, Thailand, Malaysia, Australien und Chile endemisch ist [1]. Der Erreger wird durch Milben übertragen, und die Erkrankung tritt vor allem in ländlichen Regionen und insbesondere bei Menschen, die in der Landwirtschaft arbeiten, auf [1]. Gelegentlich kommt es wie im beschriebenen Fall auch bei Reisenden zu Infektionen [2–4].

Aufgrund der unspezifischen Symptome wird hierzulande häufig nicht an *Orientia tsutsugamushi* gedacht

Klinisch kann sich das Tsutsugamushi-Fieber (auch als Milbenfleckfieber oder Scrub Typhus bezeichnet) mit einem breiten Spektrum an Symptomen präsentieren, darunter hohes Fieber, Kopfschmerzen, gastrointestinale Beschwerden, Myalgien sowie Lymphadenopathie [5]. Ein makulopapulöser Hautausschlag und ein Eschar, eine charakteristische schmerzlose Primärläsion mit schwarzer zentraler Nekrose, treten häufig auf, sind jedoch nicht obligat vorhanden [6]. Im beschriebenen Fall hatte der Patient keinen Eschar und das makulopapulöse Exanthem wurde zunächst als Arzneimittelexanthem gewertet. Aufgrund der unspezifischen Symptome wird in nichtendemischen Regionen häufig nicht an diese Differenzialdiagnose gedacht; in einer Fallserie erhielten 95 % der Patient:innen vor Diagnose eine antiinfektive Therapie, die nicht gegen *Orientia tsutsugamushi* wirksam war [7].

Schwere Verläufe können mit einer interstitiellen Pneumonie, einer Leberbeteiligung, einem akuten Nierenversagen, kardialen Manifestationen wie einer Myokarditis sowie einer Beteiligung des zentralen Nervensystems mit Meningoenzephalitis und Krampfanfällen einhergehen [5, 8, 9]. Zudem kann es zu weiteren Komplikationen, wie einem „acute respiratory distress syndrome“ (ARDS) oder zu einer nosokomialen Pneumonie kommen [9]. In unserem Fall gehen wir aufgrund der initial unauffälligen Darstellung der Lunge in der Röntgenuntersuchung und Computertomographie und dem späteren Bild einer Lobärpneumonie von einer nosokomialen Genese der Pneumonie aus. Ein Erreger konnte in bronchoskopisch gewonnenem Bronchialsekret jedoch nicht nachgewiesen werden und eine unmittelbar durch *Orientia tsutsugamushi* verursachte Pneumonie kann nicht ausgeschlossen werden.

Sowohl Azithromycin als auch Doxycyclin sind gegen *Orientia tsutsugamushi* wirksam

Sowohl Azithromycin als auch Doxycyclin sind gegen das obligat intrazelluläre Kurzstäbchen wirksam und in schweren Fällen kann eine Kombination beider Substanzen erwogen werden [10]. β‑Laktam-Antibiotika können hingegen nicht ausreichend die Zellwand eukaryotischer Zellen penetrieren; sie erreichen daher intrazellulär nur geringe Konzentrationen und sind somit nicht geeignet. Im vorliegenden Fall wurde die Therapie von Azithromycin auf Doxycyclin umgestellt, da es unter der Therapie mit Azithromycin zu keinem ausreichenden klinischen Ansprechen kam.

Basierend auf der langen Halbwertszeit von Azithromycin gehen wir davon aus, dass nach der Umstellung der Therapie zunächst ausreichende Spiegel beider Medikamente vorlagen. Eine Kombinationstherapie führten wir aufgrund des Eindrucks des initial fehlenden klinischen Ansprechens auf Azithromycin jedoch nicht fort. Unter der fortgeführten Monotherapie mit Doxycyclin kam es zu einer raschen klinischen Besserung.

Eine multizentrische, doppelt verblindete, randomisierte, kontrollierte Studie, in der eine Monotherapie mit Azithromycin, eine Monotherapie mit Doxycyclin und eine Kombinationstherapie mit beiden Präparaten verglichen wurden, zeigte keine signifikanten Unterschiede zwischen den beiden Monotherapien. In Bezug auf den kombinierten primären Endpunkt, bestehend aus Mortalität, Komplikationen und anhaltendem Fieber, war die Kombinationstherapie beiden Monotherapien überlegen, jedoch zeigte sich eine nichtsignifikant niedrigere absolute Mortalität unter einer Monotherapie mit Doxycyclin [10].

## Fazit für die Praxis

Besteht ein Fieber unklarer Genese, sollte bei Personen, die sich in Endemiegebieten aufgehalten haben, an eine Infektion mit *Orientia tsutsugamushi* gedacht werden. Eine frühzeitige Polymerase-Kettenreaktions(PCR)-Diagnostik und die zügige Einleitung einer antiinfektiven Therapie sind entscheidend, wenn schwere Verläufe mit Multiorganbeteiligung sowie sekundäre Komplikationen wie eine nosokomiale Pneumonie vermieden werden sollen.

## Data Availability

Alle dieser Arbeit zugrunde liegenden Daten sind in diesem Artikel enthalten.
